# Numerical investigating the impact of fluid properties and oscillation frequency on micro mixer efficiency

**DOI:** 10.1016/j.heliyon.2024.e41290

**Published:** 2024-12-17

**Authors:** Sajad Ghanbari, Mohammad Sefid, Mohammad Ali Sabbaghi

**Affiliations:** Department of Mechanical Engineering, Yazd University, Yazd, Iran

**Keywords:** Mixing, Micromixer, Density, Viscosity, Mixing index, Strouhal number

## Abstract

In this research, the impact of differing densities and viscosities of two dissolving fluids on their mixing efficiency, as well as the effects of various excitation frequencies on the performance of the mixer, have been examined. For this purpose, a two-dimensional microchannel equipped with an oscillating circular cylinder was used, operating within a Strouhal number range of 0.1–0.55, with an oscillation amplitude of 0.4 cylinder diameters in a direction perpendicular to the flow direction, at a blockage ratio of $\frac{1}{3}$ and Reynolds and Schmidt numbers of 50 and 10, respectively, utilizing a finite volume method based on elements. The results obtained indicate that mixing two identical fluids represents an ideal condition, wherein the highest level of mixing occurs compared to mixing two fluids with differing densities and viscosities. As the difference in viscosity and density between the two fluids increases, the level of mixing decreases, such that an increase in the logarithmic viscosity ratio up to R = 2 results in a 257.66 % reduction in mixing, and an increase in the density ratio up to S = 3 leads to a 170.08 % reduction in mixing efficiency. However, at points where the density and viscosity increase factors are equal (s = e^R^), the increase in density has a greater impact on reducing the mixing efficiency compared to the increase in viscosity. Specifically, at an increase factor of 3 for both density and viscosity, density has a 50 % greater effect on reducing mixing efficiency than viscosity. Furthermore, it is observed that when two fluids have different densities and viscosities, changes in the Strouhal number have a lesser impact on the mixing index compared to when the two fluids are identical. By creating a difference in viscosity and density between the two mixing fluids, the mixer exhibits unique and distinct performance, such that for each viscosity and density ratio, it is observed different optimal Strouhal numbers independently. This research provides a fundamental understanding of the effects of differing density and viscosity, as well as the impact of excitation frequency on the mixing efficiency of the two fluids.

## 1 Introduction

Heat and mass transfer are crucial processes that impact various aspects of modern life, from everyday household devices to sophisticated technological systems. These principles are fundamental in diverse fields, including power generation, automotive engineering, aerospace design, heating, ventilation, and air conditioning (HVAC), as well as in the cooling of electronic equipment [[1], [2], [3], [4]]. One of the most crucial processes in the field of heat transfer is the mixing process. This phenomenon plays a vital role in enhancing the efficiency of heat exchange between different substances. Mixing can be defined as any operation used to transform a non-uniform system into a uniform one. Based on scientific applications in various fields such as chemical, pharmaceutical, polymer, biomechanical and nuclear industries, the mixing and mixers conceptions hold significant importance. There are numerous ideas for mixing two fluids, one of which involves micromixers. Often, the fluids used in these processes differ in properties such as viscosity and density, and these differences, along with increasing non-uniformity, pose challenges to achieving homogeneous mixing. Mixing can occur with various combinations between gas, liquid, and solid phases, such as solid-solid, solid-liquid, liquid-liquid, and gas-liquid mixtures. Liquid-liquid mixing refers to the mixing of two miscible or immiscible liquids. The solubility depends on the properties of the substances, allowing them to blend and form a homogeneous phase. In the mixing of miscible fluids, molecular diffusion aids in achieving maximum homogeneity in the system [5,6].

Mixing typically occurs at three scales: macro mixing, meso mixing, and micromixing. Micromixing refers to the combination that occurs at the smallest scales of fluid motion and molecular movement. The increasing demands in biological, medical, chemical, agricultural and food industries have led to a significant focus on micro-mixers [7]. This is mainly due to the numerous advantages that micro-mixers offer compared to macro systems. These advantages include high reaction efficiency, elevated response sensitivity, precise control capabilities, and reduced reagent consumption. As industries continue to evolve and seek more efficient and sustainable solutions, the role of micro-mixers is becoming increasingly critical, propelling research and application in these fields forward [8]. Due to the dominance of viscous effects, turbulent flow is not feasible at micro dimensions, and for many applications, the fluid velocity at microscopic scales cannot be excessively high. The Reynolds number at these scales is usually less than 100. In microscopic domains such as microchannels, turbulent dispersion does not occur, as only laminar flow develops at low Reynolds numbers [8,9]. Considering that the characteristic length of micromixers has been reduced to the micro-scale, the surface-to-volume ratio significantly increases. This enhancement facilitates mass transfer between liquids and intensifies the fluid reaction rate. As a result, micromixers demonstrate improved efficiency in mixing and reaction processes due to the greater interaction area available for mass exchange. The micro-scale design not only optimizes the dynamics of fluid flow but also promotes a more effective and rapid blending of reagents, which is crucial in applications such as chemical synthesis, pharmaceuticals, and biochemical reactions [[10], [11], [12]]. The performance of such processes relies on the rapid and effective mixing of samples and reactants. Micro mixers are typically designed based on channel geometry to reduce mixing length and increase the contact surface area. According to these two fundamental principles used to enhance mixing at the micro-scale, micro mixers are generally divided into two categories: active (forced) and passive (non-forced). Passive micromixers facilitate enhanced mixing based on specific channel designs, while active micromixers rely on external energy sources and employ moving flow obstruction devices or pressure gradients to increase mixing [[13], [14], [15]].

Given the geometry used and the application of external energy to create forced oscillations on the obstruction body, the present research falls under the category of active micromixers. Experimental and computational studies on the harmonic oscillations of flow obstruction devices have received considerable attention in recent years [16,17]. Koch et al. [18] conducted an experimental study using flow imaging with red and green dyes dissolved in ethanol to test a horizontal micromixer. In this experiment, phenolphthalein was used, and the final mixing color depended solely on the PH level. All techniques were based on the PH indicator properties of phenolphthalein. Liu et al. [19] investigated the performance of a three-dimensional helical mixer using a phenolphthalein solution and sodium hydroxide solution in an experimental study at Reynolds numbers ranging from 6 to 70, demonstrating that mixing capabilities in the channel increased with rising Reynolds numbers. In another study, Liu et al. [20] numerically examined the mixing of two fluids, pure water and a glycerol-water solution, in two three-dimensional helical micro mixers and a zigzag mixer at Reynolds numbers of 1 and 10, identifying a critical Reynolds number (Re = 1). They observed that at this critical Reynolds number, the performance of both mixers was inversely related to the glycerol concentration. The two fluids used in the study were also employed by Jin et al. [21] to investigate the performance of a rotary mixer. They reported that as the glycerol mass ratio in water increased the level of mixing decreased. Sj et al. [22] investigated the mixing of two identical fluids in a micromixer equipped with an oscillating and forced-rotating blade. To evaluate the mixer's performance, they introduced a new mixing index suitable for time-dependent and repeatable flows, demonstrating that in all cases, the oscillating mode outperformed the rotating mode. Kim et al. [23] performed a numerical analysis of a micromixer featuring a fixed cylindrical body and an oscillating blade while mixing two identical fluids. They showed that mixing with a combination of a moving blade and a cylindrical body achieved a mixing efficiency 27 % higher than that of a system using only a moving blade. Park et al. [24], along with Ryu et al. [25], focused on the optimal design of an active micromixer with an oscillating mixer for mixing two identical fluids. To achieve effective mixing, they predicted the effective mixing index using Taguchi's method and the Kriging method utilizing a genetic algorithm. They were able to present a model that improved the micro mixer's efficiency by 85.59 % and 71.79 %, respectively, compared to the initial model. Celik and Beskok [26] investigated the mixing process of an active meso scale mixer in similar fluids using a transverse oscillating circular cylinder in a channel with Re = 100 and *Pe* = 100. They reported that the most favourable mixing condition is observed for S = 3 with a frequency ratio of F = 1.25. In their study, Zhan et al. [27] utilized computational fluid dynamics CFD to numerically evaluate the flow dynamics of highly viscous fluids being expelled from a conical container subjected to low-frequency vibrations. The researchers thoroughly examined how different rheological and vibration parameters influence the flow behavior of these fluids during the process. In the research carried out by Jing and Zhan [28], the focus is on quantitatively analyzing the pressure drop and mixing efficiency of fluid flow within a microchannel featuring a cylindrical structure and a vertically flexible flag (CCFF). This study explores how varying the height and deformability of the flag affects fluid dynamics over a broad range of Reynolds numbers based on the diameter of the cylinder. The findings reveal that the pressure drop associated with the CCFF configuration consistently exceeds that of the standard configuration (SC). However, it is noted that the mixing efficiency of the CCFF surpasses that of the SC only when the Reynolds number or the height of the flexible flag reaches sufficiently high values. In a study conducted by Shamsaddini et al. [29], an improved Incompressible Smoothed Particle Hydrodynamics (ISPH) algorithm has been introduced for simulating the mixing flow within an active micromixer that utilizes an oscillating cylinder to boost the mixing efficiency. The research explores mass transport scenarios where the inlet fluids possess varying viscosities. The findings suggest that differences in viscosity significantly influence the rate of mixing. Also, other numerical and experimental studies for different fluids have been carried out by researchers [[30], [31], [32], [33]].

Based on the existing research background, the present study distinguishes itself from other similar studies that only focus on the performance of a mixer in combining two identical fluids by examining and analyzing the mixing of two fluids with different characteristics. Furthermore, one of the notable features of this study, which sets it apart from all other research related to the mixing of two fluids, is the independent examination of the differing densities and viscosities of these fluids and their comparison with each other. This study also investigates the independent impact of these two parameters on the behavior of the mixed fluid flow and the degree of mixing achieved. This approach aims to provide a deeper understanding of how these differences affect the degree of blendability and flow behavior. To achieve this goal, a micro-mixer with an oscillating cylinder, aligned with the physics of the problem as investigated by Beskok [26] will be utilized. By making slight modifications to the previous design that addressed the mixing of two identical fluids, a range of density and viscosity ratios between the two fluids will be examined to analyze mixing behavior and the degree of blendability. Additionally, the effect of excitation frequencies when the two fluids possess different properties remains an unsolved issue. Therefore, in the continuation of this research, this topic will be investigated and compared for three different states concerning density and viscosity, as well as considering two identical fluids.

## 2 Governing equations and numerical study

Mathematical modeling of energy systems is a novel and effective approach for simulating their heat transfer performance. By employing advanced mathematical techniques, researchers and engineers can create detailed simulations that accurately represent the behavior of various energy systems under different conditions [34,35]. To simulate mixing in the active micromixer, the two-dimensional, unsteady, incompressible Navier-Stokes equations are numerically solved, along with the scalar transport equation in the described domain. In the presence of two fluids with differing densities and viscosities, mixing can be simulated using the general transport equations. In the present study, a mixed variable model is utilized to simulate single-phase multi-component flow. In this model, the proportion of each component may vary over time or with changes in location. Under these conditions, the average values of physical properties in each control volume are calculated, which depend on the properties and proportions of each component present in that volume. In multi-component flows, it is assumed that the components are well-mixed at the molecular level, and the average values of velocity, pressure, and temperature are also considered uniform. Additionally, mass transfer in these flows occurs through advection and diffusion processes. The mixing of two fluid streams is considered with the following general transport equations [20,26,[36], [37], [38]]:

(1) $$\frac{\partial \rho C}{\partial t}+\nabla ∙\left(\rho \vec{V}C\right)=\nabla ∙\left(\rho D\nabla C\right)$$

Where ρ, C, D, and $\vec{V}$, represent the density of the mixture, concentration, mass diffusion, and velocity vector, respectively. For most pairs of mixing fluids, a density-concentration relationship provides a good approximation: $\rho =\rho_{1}(1+C(S-1$) [35,36] where $S=\rho_{2}/\rho_{1}$ is the density ratio. Subscripts 1 and 2 indicate the first and second fluids, respectively.

The continuity, momentum equations, and shear stress are defined as follows [35,36]:

(2) $$\frac{\partial \rho }{\partial t}+\nabla ∙\left(\rho \vec{V}\right)=0$$

(3) $$\frac{\partial \rho \vec{V}}{\partial t}+\nabla ∙\left(\rho \vec{V}\vec{V}\right)=-\nabla p+\nabla ∙\left(\vec{\tau }\right)$$

(4) $$\vec{\tau }=\mu \left[\left(\nabla \vec{V}+{\left(\nabla \vec{V}\right)}^{T}\right)-\frac{2}{3}\delta \left(\nabla .\vec{V}\right)\right]$$

**Fig. 1:**
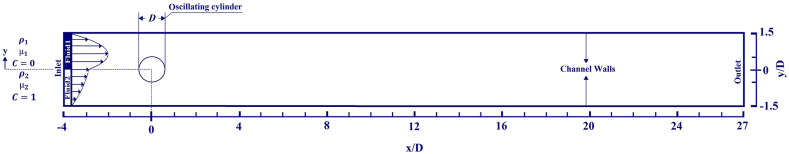
Schematic of the oscillating cylinder micromixer in the mixing of the two different fluids.

The interaction of miscible fluids may lead to a wide spectrum of viscosity-concentration behaviors, which can exhibit characteristics ranging from exponential trends to non-linear patterns. As a result, previous numerical studies have utilized various models to describe these relationships. Following previous research [[34], [35], [36],[39], [40], [41]], it is assumed that the viscosity μ is an exponential function of concentration, as initially introduced by Tan [42]: $\mu =\mu_{1}e^{CR}\text{.}$ Where $R=\ln\;\mu_{2}/\mu_{1}$ is the logarithmic dynamic viscosity ratio. The control volume method is employed in solving computational fluid dynamics. These equations are integrated over each control volume, and Gauss's divergence theory is applied to convert volume integrals involving divergence and gradient operators into surface integrals. The integral equations must be modified when control volumes deform over time. These modifications arise from the application of the Leibniz rule. The geometry of the micromixer is depicted in Fig. 1. The height of the microchannel is set at 3D, and its length is 31D. A circular cylinder with a diameter of D is considered, with its centre located 4D from the channel inlet. The centre of the cylinder aligns with the centre of the channel, and oscillation occurs in the y-direction as follows:

(5) $$y=y_{\max}\;\sin\left(\omega t\right)$$

Where, y_max_ and ω refer to the maximum cylinder displacement, and angular velocity, respectively. In the current study, y_max_ is considered as a fraction of 0.4 of the cylinder diameter (y_max_ = 0.4D).

In all cases, the characteristics of the first fluid are constant, while for the second fluid, only the density and viscosity have changed. The first fluid enters the channel from the upper half, and the second fluid enters from the lower half. The most important dimensionless parameters in this research are the Reynolds number (Re), Peclet number (*Pe*), and Strouhal number (St), which are defined as follows based on the first fluid:

(6) $$Re=\frac{\rho_{1}uh}{\mu_{1}}$$

(7) $$Pe=\frac{hu}{d}=Re\;Sc$$

(8) $$St=\frac{fD}{u}$$

Here, u, h, D, f, d, and Sc represent the average fluid velocity, channel inlet height, cylinder diameter, forced oscillation frequency, mass diffusion, and Schmidt number, respectively. In this study, no-slip boundary conditions are considered at the surface of the cylinder as well as at the channel walls. Zero Neumann boundary conditions are applied at all these boundaries for the scalar transport equations. At the outlet, zero Neumann conditions are employed for both velocity and species concentration. For the two species entering the channel, C = 1.0 is applied for y > 0 and C = 0.0 for y < 0.

The inlet velocity is applied as a developed profile. Since the density and viscosity of the two fluids are different, the developed velocity profile changes with varying viscosity. Therefore, a developed velocity profile for two immiscible fluids is applied at the inlet. When only the density differs (with constant viscosity), the developed velocity profile remains unchanged with varying density due to zero acceleration and the removal of density (inertial term) from the linear momentum equation. However, this is not the case for viscosity, as viscosity affects the developed velocity profile. The following equation defines the developed velocity profile for two immiscible fluids with differing viscosities for a channel with an inlet height, h [34,43].

(9) $$\left(y\right)=\left\{ \begin{array}{c} U_{0}\left(1+A\frac{y}{h/2}-B{\left(\frac{y}{h/2}\right)}^{2}\right),y\geq 0\\ U_{0}\left(1+\overset{´}{A}\frac{y}{h/2}-\overset{´}{B}{\left(\frac{y}{h/2}\right)}^{2}\right),y\leq 0 \end{array}\right.$$

(10) $$A=\frac{\mu_{2}-\mu_{1}}{2\mu_{1}}$$

(11) $$B=\frac{\mu_{2}+\mu_{1}}{2\mu_{1}}$$

(12) $$\overset{´}{A}=\frac{\mu_{2}-\mu_{1}}{2\mu_{2}}$$

(13) $$\overset{´}{B}=\frac{\mu_{2}+\mu_{1}}{2\mu_{2}}$$

(14) $$U_{0}=\frac{2u}{E}$$

(15) $$E=2+\left(\frac{A-\overset{´}{A}}{2}-\frac{B-\overset{´}{B}}{3}\right)$$

In the present study, the mixing index, M, is used to quantify the mixing efficiency of the mixer and is defined as a measure of deviation from complete mixing as follows [44]:

(16) $$M=\sqrt{\frac{1}{N}\sum_{i=1}^{N}{\left(\frac{C_{i}-C_{\text{mean}}}{C_{\text{mean}}}\right)}^{2}}$$

N denotes the number of points in a channel cross-section, Ci is the concentration at each point, and Cmean is the average concentration, which is 0.5. This index ranges from 0 to 1. In the case of complete mixing, the value of this index approaches zero, while when no mixing occurs, the index equals 1. A value of less than 0.2 indicates that sufficient mixing has taken place [26].

In the present case, where the flow is time-dependent, it is necessary to calculate the mixing index for oscillation. The time-dependent mixing index is expressed as follows:

(17) $$DI=\frac{1}{T}\int_{T}^{.}M\;dt$$

Where, T is the sampling time interval, which corresponds to one or several periods of the blade oscillation. The percentage reduction in mixing efficiency (η) for the mixing index at the outlet of the channel (DIout) relative to the baseline mixing index (DIst) is defined as follows:

(18) $$\eta =\frac{{DI}_{\text{st}}-{DI}_{\text{out}}}{{DI}_{\text{st}}}\times 100\%$$

The baseline mixing index (DIst) refers to the condition in which to achieve the best possible mixing. Accordingly, by comparing this baseline index with the mixing index at the outlet of the channel (DIout), the percentage reduction in mixing efficiency (η) is determined. A positive η indicates that the mixing is less effective compared to the baseline condition, with larger values of η signifying greater reductions in mixing efficiency. According to previous definitions, the Reynolds number and Schmidt number are set at 50 and 10, respectively. Additionally, the Strouhal number, density ratio, and logarithmic viscosity ratio are considered within the ranges 0.1≤ St ≤ 0.55, 1≤ S ≤ 3, and 0≤ R ≤ 2.

## 3 Results and discussions

### 3.1 Grid study

**Fig. 2:**
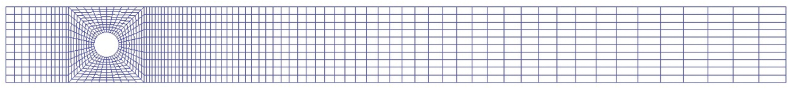
Microchannel equipped with oscillating cylinder computational grid.

**Fig. 3:**
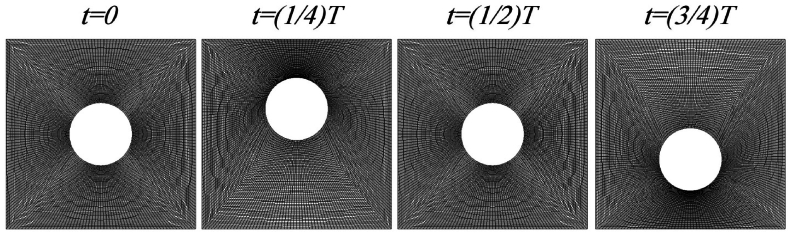
Computational grid for oscillating cylinder during full period.

In this study, a dynamic mesh is considered over the complete oscillation period (T). Fig. 2, Fig. 3 illustrate the computational grid used and the computational mesh throughout the full cycle, respectively.

**Fig. 4:**
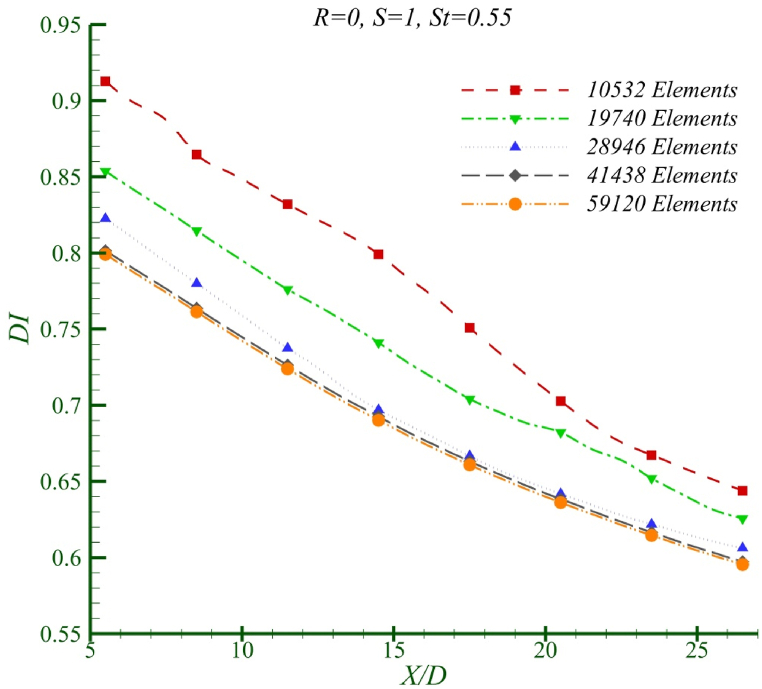
The effect of the grid size in Re = 50, St = 0.55 for two similar fluids.

Fig. 4 shows the effect of grid size at Re = 50 and St = 0.55 for two similar fluids. As observed with 41,438 and 59,120 grid elements, the difference in the mixing index results is less than 1 %. Therefore, the grid with 41,438 elements has been selected as the final grid for the computations.

There are several mesh quality metrics, some of the most critical parameters are orthogonality, aspect ratio and skewness [45]. More details about the meshing and solution of the studied geometry are presented in Table 1.

**Table 1:** Detailed meshing for the investigated geometry.

Parameter	Minimum	Maximum	Average
**Orthogonal Metric**	0.70867	1	0.917149
**Aspect Ratio**	1.0971	7.4545	2.6786
**Skewness**	1.0357e-010	0.5	8.0061e-002

### 3.2 Validation

**Fig. 5:**
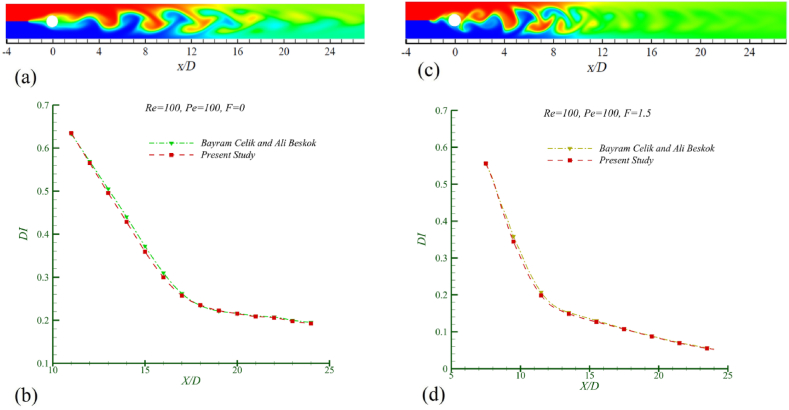
The validation of the present study in F = 0 a) Concentration contour b) Mixing index and in F = 1.5 c) Concentration contour d) Mixing index.

The research conducted by Beskok et al. [26], which is the closest work to the physics of the present study, was validated at two frequency ratios $\left(F=f_{e}/f_{0}\right)$ of 0 and 1.5, at Re = 100 and *Pe* = 100. Fig. 5 illustrates the results of this validation for both cases, where the difference in mixing index outcomes is less than 1.5 %. The boundary conditions and the physics of the current study have been considered similar to Beskok's research, in terms of the dimensions and oscillation amplitude of the cylinder. However, Beskok had placed a separating plate at the beginning of the channel that divided the two fluids. The authors decided to remove this separating plate at the channel entrance, based on the implementation of a developed velocity profile at the channel's start. The most significant aspect of the problem pertains to the properties of the mixing fluids. Beskok examined the mixing of two identical fluids while focusing on the effects of excitation frequency and the Peclet number. However, in the present study, the authors moved beyond these ideal conditions to align with real-world scenarios by investigating the mixing of two fluids with differing viscosity and density characteristics at various oscillating excitation frequencies. As will be demonstrated in the results, this difference reveals that mixing two fluids with distinct properties during the blending process has significantly different impacts on the performance of the mixer and the behavior of the mixed fluid flow, compared to the ideal case of two identical fluids.

### 3.3 Results and discussion

In the numerical analysis, the impact of differing densities and viscosities of two dissolving fluids on their mixing efficiency, considering five different ratios for each fluid is investigated. Since mixing in this channel is achieved through the vortex formation mechanism behind the cylinder, this operation occurs with a forced excitation frequency across 10 different Strouhal numbers, with oscillation of the cylinder taking place within the transverse channel range of −0.4D ≤ y ≤ 0.4D. The mixing behavior and the mixer are examined in two main categories.

A) The impact of differing viscosity and density on mixing at a constant Strouhal number:
• Mixing of two fluids with different viscosities and identical densities (S = 1, 0≤ R ≤ 2) at a Strouhal number of 0.35.
• Mixing of two fluids with different densities and identical viscosities (1≤ S ≤ 3, R = 0) at a Strouhal number of 0.35.
B) The effect of Strouhal number in three scenarios:
• Mixing of two identical fluids (S = 1, R = 0) at various excitation frequencies 0.1≤ St ≤ 0.55.
• Mixing of two fluids with different viscosities and identical densities (S = 2.5, R = 0) at various excitation frequencies 0.1≤ St ≤ 0.55.
  • Mixing of two fluids with different viscosities and densities (S = 1, R = 1.5) at various excitation frequencies 0.1≤ St ≤ 0.55.

#### 3.3.1 Effect of different viscosity and density on the mixing process

**Fig. 6:**
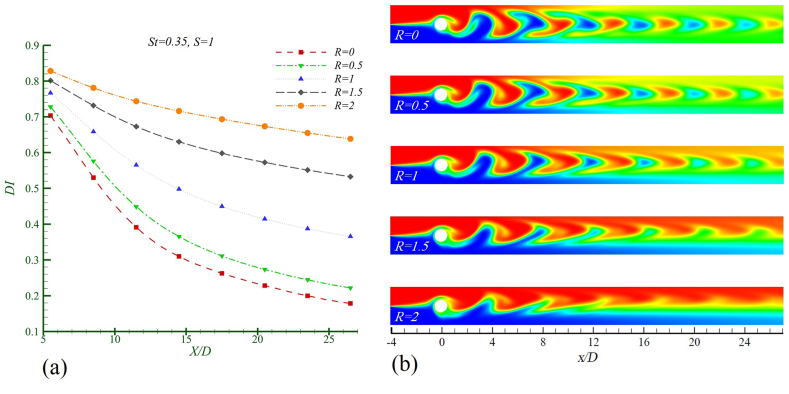
Mixing in Re = 50, Sc = 10, St = 0.35 and S = 1 in different logarithmic viscosity ratios in a)mixing index b)concentration contour.

To investigate the effect of increasing the viscosity ratio on mixing behavior and efficiency, the Re = 50, Sc = 10, and St = 0.35 with five viscosity ratios R = 0, 0.5, 1, 1.5, and 2 while maintaining the same density (S = 1) is considered. In Fig. 6a, the mixing index is presented, and Fig. 6b shows the concentration contour from this analysis. As the fluid flows past the cylinder, a Karman vortex street forms behind the oscillating cylinder extending to the end of the channel. The primary mixing operation is related to the number and intensity of the vortices shed from the cylinder.

As seen in the concentration contour in Fig. 6b, at R = 0 (where the two fluids are identical), a Karman vortex street is formed with stronger vortex intensities compared to cases where R > 0. As the difference in viscosity increases (with a larger R), the Karman vortex street exhibits weaker vortex intensities. In the case of R = 2, the fluid struggles to form suitable vortices, leading to the initial vortices dissipating for X/D > 12, resulting in a loss of the primary mixing capability.

The increase in the size of the light-colored area indicates improved mixing. However, with a greater difference in the viscosities of the two fluids, the fluid becomes resistant to deformation, leading to the formation of poorly mixed vortex centres. These vortices exhibit less lateral growth, causing the bright-colored region to be limited to the middle section of the channel where the two fluids meet. Near the walls, as R increases, this region becomes significantly smaller.

The mixing index DI in Fig. 6a, which reflects the mixing efficiency during one oscillation period of the cylinder, shows that when the two fluids are identical, the highest mixing efficiency occurs at the outlet of the channel. As R increases, this index rises, indicating a decrease in mixing efficiency. Furthermore, with a larger R, the range of variation in this index becomes limited, such that as R increases from 0 to 2, the oscillation range of DI in the region (X/D > 5) decreases from 0.18 to 0.7 to 0.64−0.83.

As shown in Table 2, the baseline mixing index DIst (the optimal mixing condition) for the case of two identical fluids (R = 0) is considered. It is observed that as R increases from 0 to 2, the rate of decrease in mixing efficiency increases by 257.66 %.

**Table 2:** The rate of decreasing of the amount of mixing compared with the base mixing index at channel outlet for Re = 50, Sc = 10, St = 0.35 and S = 1 in various viscosities.

logarithmic viscosity ratio	R = 0	R = 0.5	R = 1	R = 1.5	R = 2
*η* (%)	0	24.27	104.81	198.39	257.66
Mixing index changes	Base (no change)	Decreasing	Decreasing	Decreasing	Decreasing

The further increase in the viscosity of fluid number 2 results in greater viscous forces and increased resistance of the fluid to external forces. This leads to enhanced heterogeneity between the two fluids and hinders the shedding of strong vortices from the cylinder, consequently resulting in a decrease in the mixing efficiency of the two fluids.

**Fig. 7:**
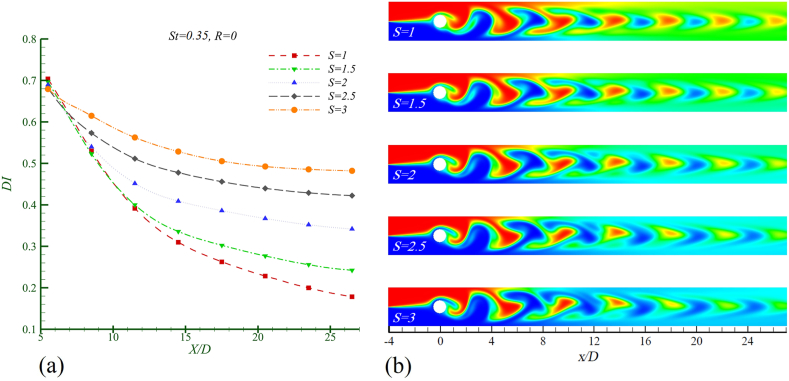
Mixing in Re = 50, Sc = 10, St = 0.35 and R = 0 in different density ratio in a)mixing index b)concentration contour.

In Fig. 7a, the mixing index is plotted, and Fig. 7b shows the concentration contour of two fluids considering Re = 50, Sc = 10, and St = 0.35 across five density ratios S = 1, 1.5, 2, 2.5, and 3 with constant viscosity (R = 0). The mixing index graph (Fig. 7a) indicates that as the density of fluid 2 increases, the mixing index also increases, which signifies a reduction in mixing efficiency. In the case of two identical fluids, the mixing index reaches its lowest value, indicating the highest mixing efficiency at the channel outlet. Similar to the effect of increasing viscosity differences, the mixing index graph (Fig. 7a) shows that as S increases, the range of variation in this index becomes limited, reducing the oscillation range of DI in the region (X/D > 5) from 0.18 to 0.7 to 0.48−0.68 as S increases from 1 to 3.

In the concentration contour (Fig. 7b), it is observed that with increasing S, the size of the light-colored area diminishes. This reduction clearly indicates a decrease in mixing efficiency and a poorer performance of the mixer. Unlike the observed effect of viscosity, where increasing R led to a reduction in vortex shedding, the increase in S does not reduce vortex shedding but instead decreases fluid dispersion and molecular diffusion, resulting in reduced mixing efficiency. As the vortices shed from the cylinder for larger S values, the “vortex core centered poor mixing zone” becomes more pronounced. Consequently, the fluid trapped in these vortices does not disperse adequately along the channel, leading to decreased mixer performance and lower mixing efficiency.

In Table 3, the baseline mixing index DIst (the optimal mixing condition) is considered for the case of two identical fluids (R = 0). It is observed that as S increases from 1 to 3, the rate of decrease in mixing efficiency grows by 170.08 %.

**Table 3:** The rate of decreasing of the amount of mixing compared with the base mixing index at channel outlet for Re = 50, Sc = 10, St = 0.35 and R = 0 in various densities.

Density rate	S = 1	S = 1.5	S = 2	S = 2.5	S = 3
*η* (%)	0	35.71	91.52	136.56	170.08
Mixing index changes	Base (no change)	Decreasing	Decreasing	Decreasing	Decreasing

The further increase in the density of fluid number 2 leads to a higher amount of fluid being trapped in the vortex cores, increasing the heterogeneity between the two fluids. This results in greater resistance to the dispersion of the trapped fluid, which ultimately leads to a decrease in the mixing efficiency of the two fluids.

Although none of the previous studies have examined the independent effects of viscosity and density, or compared these two factors and their influence on mixing, it was observed in both the viscosity and density sections that the performance of the mixer has an inverse relationship with increasing S and R. This is consistent with the findings of Liu et al. [20] who, in their research on the mixing of water and a glycerin-water mixture, indicated that the performance of the mixer inversely correlates with the mass fraction of glycerin in pure water, due to the dominance of molecular diffusion.

**Fig. 8:**
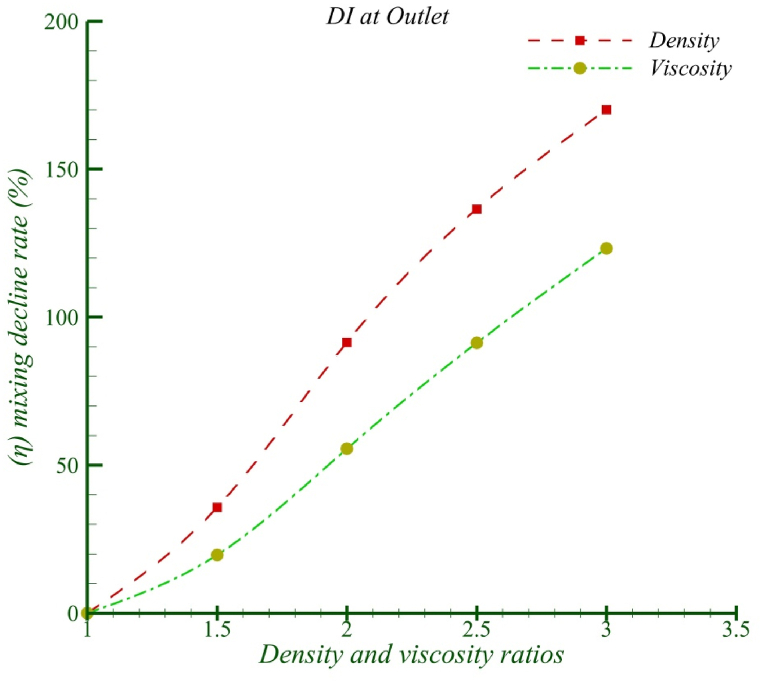
Change of mixing decline rate in different density and viscosity ratios.

To answer the question of whether the increase in density or viscosity of fluid 2 has a greater impact on the reduction in mixing efficiency, it is necessary to compare the simple viscosity ratio instead of the exponential viscosity at equal density ratios (s = $e^{R}=\frac{\mu_{2}}{\mu_{1}}=\frac{\rho_{2}}{\rho_{1}}$). As shown in Fig. 8, at corresponding density ratios, it is evident that density has a more significant effect on the reduction in mixing efficiency. Specifically, at a ratio of 3, the density has a 50 % greater impact on reducing mixing efficiency compared to viscosity.

#### 3.3.2 Effect of Strouhal number on the mixing process

To investigate the effect of excitation frequency on mixing performance, the simulations considering Re = 50, Sc = 10 for three different scenarios are conducted: two identical fluids (S = 1, R = 0), two fluids with a density ratio of 2.5, and two fluids with a logarithmic viscosity ratio of 1.5, across 10 different Strouhal numbers ranging from 0.1 to 0.55 in increments of 0.05. The average species concentration at the channel inlet was maintained at 0.5 for all analyses.

**Fig. 9:**
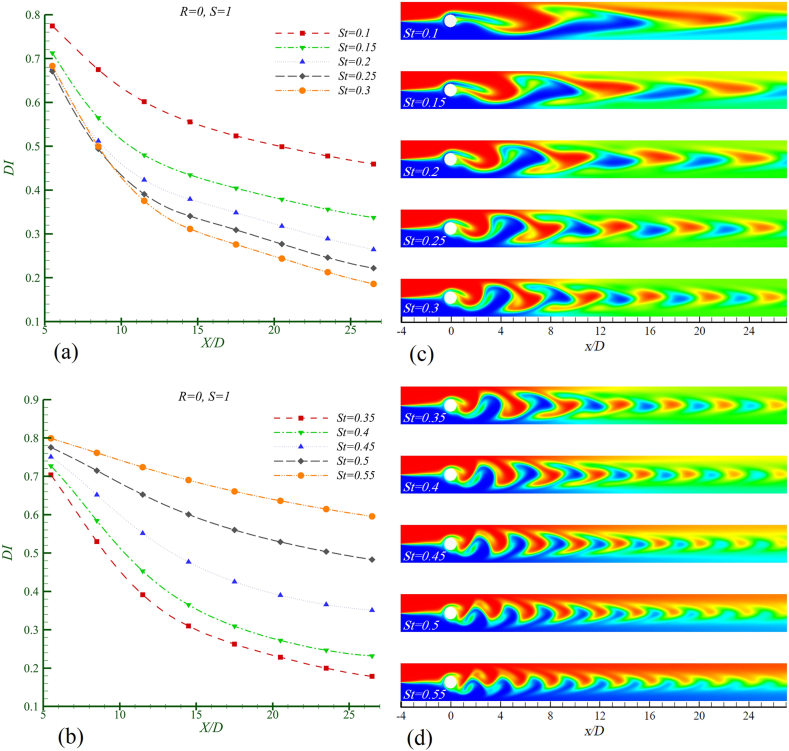
Mixing in Re = 50, Sc = 10, S = 1, R = 0 in different Strouhal number. a) mixing index, b) concentration contour.

All figures depict the instantaneous concentration contour as the cylinder moves upward from the position y = 0 at t = 0. In Fig. 9a and b, the mixing index is presented, while Fig. 9c and d shows the concentration contours for the two identical fluids at different Strouhal numbers. As observed from the mixing index graph, the most efficient mixing occurs at St = 0.35. With an increase in the excitation frequency of the cylinder, the distance between the shed vortices decreases while both their number and intensity increase. However, an optimal mixing scenario requires a suitable combination of enhanced lateral growth of the vortices alongside increased intensity and reduced spacing between them; this is most evident at St = 0.35. The fluid trapped in the vortices at this Strouhal number rapidly mixes due to the increased strength of the vortices and appropriate lateral growth upon contacting the channel walls, thereby improving overall mixing. At St < 0.35, despite the suitable lateral growth of the vortices, inadequate vortex strength results in suboptimal mixing. Conversely, at St > 0.35, although the vortex strength is adequate, insufficient lateral growth leads to a decline in mixing efficiency, resulting in a more pronounced vortex core centered poor mixing zone in both cases, with a reduction in the size of the light-colored area.

According to Table 4, the baseline mixing index DIst (optimal mixing condition) at St = 0.35 is considered. It is observed that as St increases from 0.35 to 0.55, the rate of decrease in mixing efficiency rises by 233.6 %. Conversely, as St decreases from 0.35 to 0.1, the rate of decrease in mixing efficiency increases by 157.28 %. The obtained result can be aligned with the fluid flow observed in the findings presented by Bescok [26]. Initially, an increase in the stimulation frequency leads to a rise in the level of mixing. However, after reaching a critical frequency, any further increase in the stimulation frequency negatively impacts the mixing rate. This indicates a complex relationship between stimulation frequency and mixing efficiency, highlighting the importance of identifying optimal conditions for achieving the desired level of fluid interaction.

**Table 4:** The rate of decreasing of the amount of mixing compared with the base mixing index at the channel outlet in Re = 50, Sc = 10, R = 0 and S = 1.

**Strouhal number**	**St** = **0.1**	**St** = **0.15**	**St** = **0.2**	**St** = **0.25**	**St** = **0.3**
*η* (%)	157.28	88.81	48.09	24.24	4.19
Mixing index changes	Decreasing	Decreasing	Decreasing	Decreasing	Decreasing

Strouhal number	St = 0.35	St = 0.4	St = 0.45	St = 0.5	St = 0.55

*η* (%)	0	29.85	96.59	170.58	233.6
Mixing index changes	Base (no change)	Decreasing	Decreasing	Decreasing	Decreasing

**Fig. 10:**
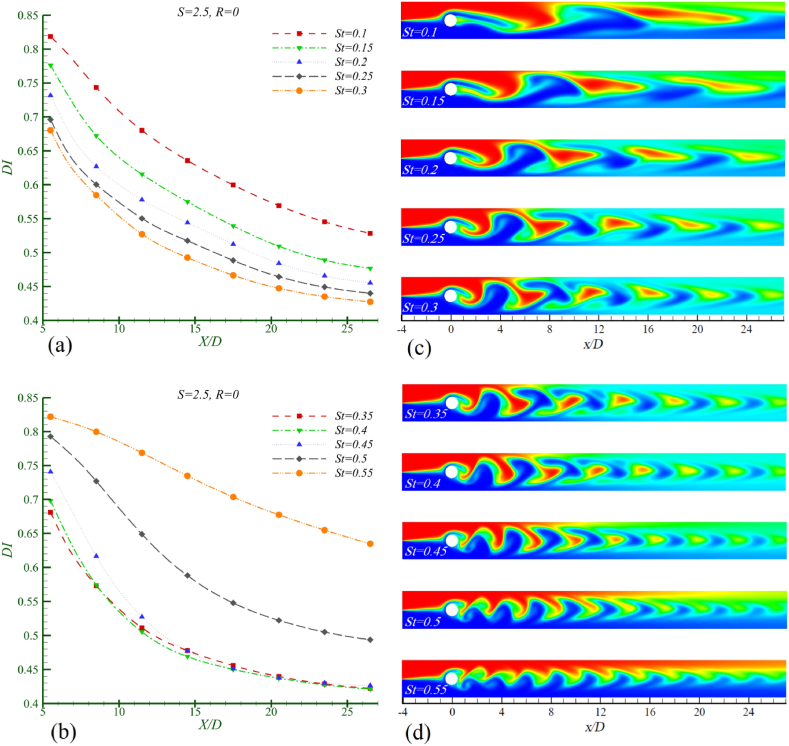
Mixing in Re = 50, Sc = 10, S = 2.5, R = 0 in different Strouhal numbers, in a,b) mixing index c,d) concentration contour.

Fig. 10a and b displays the mixing index, while Figs. 10c and d illustrate the concentration contours, showcasing the effect of the Strouhal number on mixing and the performance of the mixer for two fluids with a density ratio of 2.5. As the Strouhal number increases to 0.40, an improvement in mixing performance is observed. However, as the Strouhal number exceeds 0.40, a further decrease in mixing efficiency occurs. By comparing the concentration contours at different Strouhal numbers, it is evident that the best mixer performance occurs when there is a suitable combination of enhanced lateral growth of the vortices, increased vortex intensity, and reduced spacing between them. Nonetheless, qualitative comparisons of the concentration contour images do not provide a definitive measure of the most efficient mixing. To determine the optimal mixing condition, it is necessary to determine the mixing index. Upon examining the mixing index graphs and comparing them in Table 5, it is shown that at St = 0.4, the performance is slightly better than at the adjacent Strouhal numbers. Considering St = 0.4 as the baseline mixing index DIst (optimal mixing condition), it is observed that as St increases from 0.4 to 0.55, the rate of decrease in mixing efficiency rises by 50.67 %. Conversely, as St decreases from 0.4 to 0.1, the rate of decrease in mixing efficiency increases by 23.59 %.

**Table 5:** The rate of decreasing of the amount of mixing compared with the base mixing index at the channel outlet in Re = 50, Sc = 10, R = 0 and S = 2.5.

Strouhal number	St = 0.1	St = 0.15	St = 0.2	St = 0.25	St = 0.3
*η* (%)	25.39	13.15	8.04	4.44	1.47
Mixing index changes	Decreasing	Decreasing	Decreasing	Decreasing	Decreasing

Strouhal number	St = 0.35	St = 0.4	St = 0.45	St = 0.5	St = 0.55

*η* (%)	0.24	0	1.23	17.2	50.67
Mixing index changes	Decreasing	Base (no change)	Decreasing	Decreasing	Decreasing

**Fig. 11:**
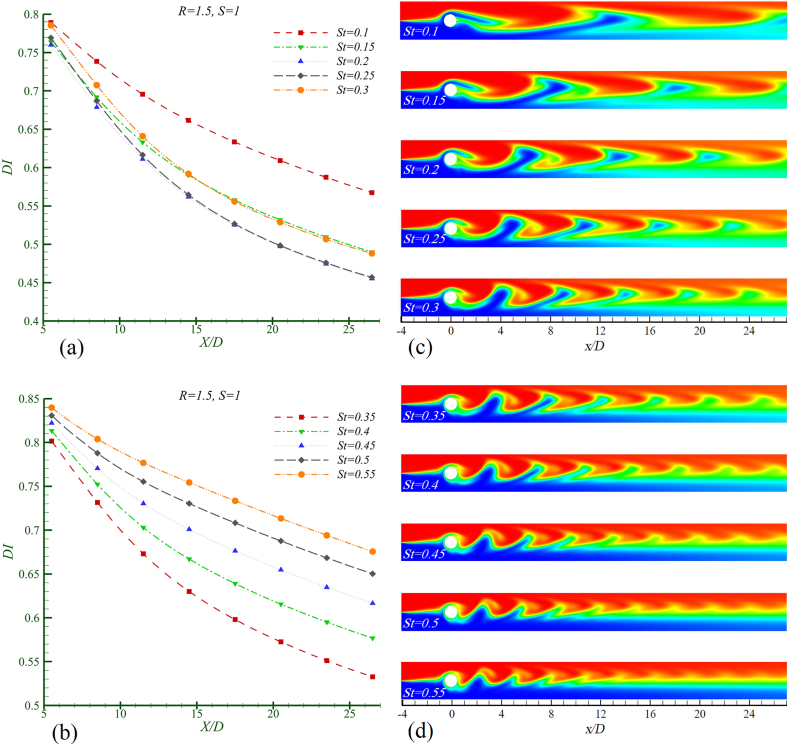
Mixing in Re = 50, Sc = 10, R = 1.5, S = 1 in different Strouhal number 0.1 ≤ St ≤ 0.55 in a,b) mixing index c,d) concentration contour.

By examining the effect of the Strouhal number on the mixing of two fluids with a logarithmic viscosity ratio of 1.5 and analyzing the mixing index graphs in Fig. 11a and b, as well as the concentration contours in Fig. 11c and d, the optimal mixing occurs at a Strouhal number of 0.2. Observing the concentration contours, it can be found that increasing St beyond 0.2 does not positively influence the lateral growth of the vortices shed from the cylinder nor enhance their strength. Furthermore, as St increases further, the lateral growth decreases sharply, and the fluid heterogeneity restricts the bright-colored region, confining it to the middle section of the channel at the interface of the two fluids. Considering St = 0.2 as the baseline mixing index ${DI}_{st}$ (the optimal mixing state), and from Table 6 is found that with an increase in St from 0.2 to 0.55, the rate of decrease in the mixing level reaches 24.63 %, while a decrease in St from 0.2 to 0.1 results in an increase of up to 48.41 %.

**Table 6:** The rate of decreasing the amount of mixing compared with the base mixing index at the channel outlet in Re = 50, Sc = 10, R = 1.5 and S = 1.

Strouhal number	St = 0.1	St = 0.15	St = 0.2	St = 0.25	St = 0.3
*η* (percent)	24.63	7.67	0	0.37	7.25
Mixing index changes	Decreasing	Decreasing	Decreasing	Decreasing	Decreasing

Strouhal number	St = 0.35	St = 0.4	St = 0.45	St = 0.5	St = 0.55

*η* (percent)	17.01	26.7	35.43	42.85	48.41
Mixing index changes	Base (no change)	Decreasing	Decreasing	Decreasing	Decreasing

**Fig. 12:**
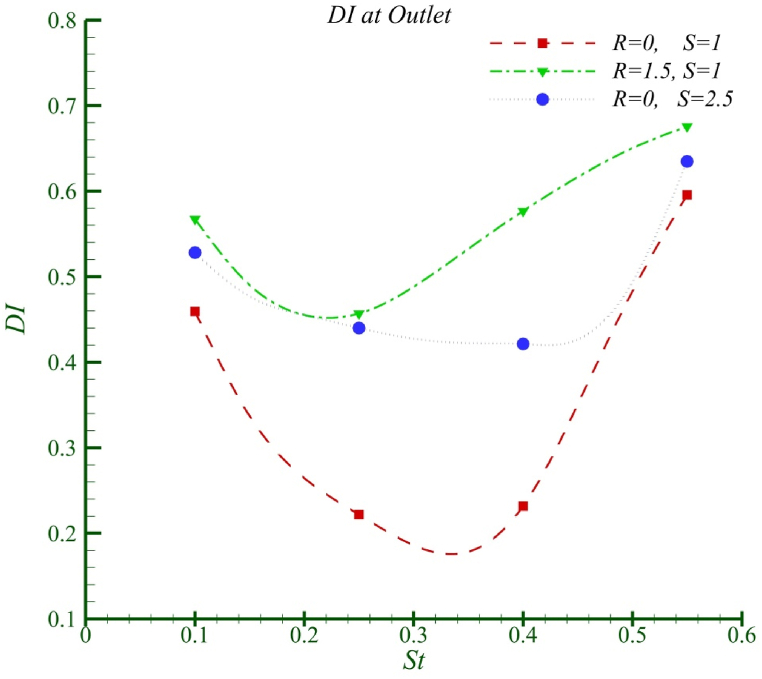
Mixing index in channel outlet.

Fig. 12 illustrates the mixing index at the outlet of the channel for the three scenarios. It shows that as the properties of the two fluids differ, the performance of the mixer changes, with each scenario exhibiting unique behaviour. Different excitation frequencies have a greater impact on mixing efficiency in the case of two identical fluids, while the range of variation in the mixing index for fluids with different densities and viscosities significantly decreases, making them less sensitive to the excitation frequency. Additionally, the optimal Strouhal number varies for each scenario. However, mixing two identical fluids represents an ideal condition, and the concept of mixing is more meaningful when the fluids have different temperatures, concentrations, or properties, rather than when they are identical.

Given the fundamental nature of mixing, it seems more logical to focus research on the performance of mixers with fluids that have different properties, rather than on identical fluids. Therefore, based on the findings of this study, using optimal points or optimized mixers designed for mixing identical fluids may not be suitable for mixing fluids with different properties. It is essential to consider the impact of varying properties of the mixing fluids on the unique behaviour of mixing and the mixer design for optimization independently.

## 4 Conclusion

In the present study, the effect of differing densities and viscosities of the two mixing fluids on the performance of a two-dimensional micro-mixer equipped with an oscillating cylinder was investigated. Given that few studies have investigated the behaviour of mixing flows with variations in the properties of two fluids and that most previous research has focused on the performance of mixers involving identical fluids, the primary distinguishing feature of the current study lies in examining the independent effects of differing densities as well as viscosities of the two mixing fluids. This study aims to analyze how these differences influence the behaviour of the mixing flow, the phenomena governing it, and the overall degree of mixing achieved. By doing so, it seeks to establish a fundamental understanding of how fluid properties impact the mixing process, thus enriching the body of knowledge in this area. The impact of various excitation frequencies based on the differing properties of the mixing fluids was also examined. The Reynolds number and Schmidt number were set at 50 and 10, respectively. The logarithmic viscosity ratio and viscosity ratio were considered within the ranges 0≤ R ≤ 2 and 1≤ S ≤ 3. The oscillation amplitude of the cylinder was set at ±0.4D, with Strouhal numbers ranging from 0.1≤ St ≤ 0.55. The conducted study revealed that as the differences in density and viscosity of the two mixing fluids increase, the performance of the mixer and the mixing efficiency decrease correspondingly. However, the difference in density has a greater impact on the reduction of mixing efficiency compared to viscosity when considering equal ratios. The differing densities and viscosities of the two fluids affect the mixer performance and the flow dynamics within the channel. Mixing two fluids with identical properties represents an ideal scenario where the mixer performs optimally, and the mixing index reaches its best state. In contrast, when the densities and viscosities differ, the Strouhal number has a lesser effect on changing the mixing index compared to the case of identical fluids.

The range of variation in the mixing index is greater when the two fluids are identical than when their densities and viscosities differ. Additionally, changes in the viscosity and density ratios lead to unique and varying behaviours in vortex shedding, vortex intensity, lateral growth, and spacing of the vortices. Each ratio corresponds to different optimal Strouhal numbers, highlighting the complexity of the mixing process under varying fluid properties.

### 4.1 Comparison with previous research

In comparison to the research conducted by Kim et al. [23], it is observed that they employed a long channel (45D) with a Reynolds number of 80 and a Schmidt number of 10 to achieve optimal mixing. Additionally, they equipped this channel with oscillating cylinders and blades. However, the results indicate that they reached a value of DI = 0.18 over 45D, whereas the present study, under its specific conditions, achieved the same value of DI = 0.18 using only a single oscillating cylinder, at a Reynolds number of 50 and a Schmidt number of 10, in a shorter channel 31D.

### 4.2 Future studies

The necessity of research and a focus on realistic conditions in the issue of mixing is crucial for achieving more applicable results. It is suggested that future studies in this field continue by examining the mixing of two fluids that have different viscosity and density simultaneously. Additionally, the impact of a heat source and its effect on the degree of mixing and the behaviour of the blending flow should be investigated in line with the current research. This approach could lead to improvements in industrial processes and the design of more efficient systems.

### 4.3 Limitation

Although the design, operation, and maintenance of active micromixers in microfluidic systems present more challenges compared to macro systems, advancements in this technology may lead to broader utilization of the advantages and applications of micromixers in the future. These micromixers are capable of executing more complex processes at the micro scale, which allows them to play a key role in various industries, including the chemical, pharmaceutical, and biotechnology sectors. With the anticipated increase in research and development in this field, significant improvements in the efficiency and performance of these systems are expected, paving the way for new applications and innovations.

**Table:** Nomenclature

Sstrouhal number	St		***Symbols***
Sampling interval time	T	Concentration	C
Average channel velocity	u	Mass diffusivity	d
Velocity at y = 0	$U_{0}$	Cylinder Diameter	D
axial coordinate	x	Mixing index (time-dependent)	DI
displacement in y direction	y	Oscillation cylinder frequency	f
	***Greek symbols***	Excitation frequency	$f_{e}$
Dynamic viscosity	μ	Natural vortex shedding frequency	$f_{0}$
Density	ρ	height of channel inlet	h
increasing or decreasing of the mixing (percent)	η	Mixing index (time-independent)	M
		number of discretized points on the cross-section for the channel	N
	***Subscript***		
Fluid1	1	Pressure	P
Fluid 2	2	Peclet number	Pe
The mean value	mean	logarithmic ratio of the viscosity	R
The channel outlet	out	Reynolds number	Re
base	st	Density ratio	S
Each point	i	Schmidt number	Sc

## CRediT authorship contribution statement

**Sajad Ghanbari:** Writing – original draft, Software, Methodology, Investigation, Formal analysis, Data curation. **Mohammad Sefid:** Writing – review & editing, Supervision, Conceptualization. **Mohammad Ali Sabbaghi:** Writing – review & editing, Visualization, Supervision, Resources.

## Declaration of competing interest

The authors declare that they have no known competing financial interests or personal relationships that could have appeared to influence the work reported in this paper.
